# Instruments

**Published:** 1868-10

**Authors:** 


### INSTRUMENTS.

We have heretofore, occasionally intimated that some improvement in the instruments employed in the operation of filling teeth was desirable. We are glad to note that the fact of needed improvement has been recognized and acted upon to a very commendable extent.

We have been using for several months (long enough to make a very thorough test) excavators and drills manufactured by S. S. White superior to anything of the kind we have before used. Their excellence consists in precision of form, excellence of temper and fineness of finish, and made of the very finest material.

There is also a very marked improvement in the plugging instruments, these being very accurately serrated and beautifully finished; the finish of the serrations is especially worthy of notice, though the points of these instruments, constitute the most important part, yet the others should not be neglected. The shaft, or that part extending from that point to the handle, should be neatly and delicately formed, and have a spring temper and be highly polished. The handles should also be of such form as to best facilitate their use in their respective work. If of steel, finely polished, silvered or gilded.

The steel handles as formerly from the forge, or the coarse file, indicates a want of that delicate taste and refinement that should always characterize the Dentist.

We have also recently obtained from H. R. SHERWOOD, of this city, some very fine excavators and bur drills; these have an excellence of finish hardly surpassed by any; the form, finish and temper of the points are most excellent. The bulbs of the bur drills were turned in a lathe (according to a suggestion we made several years ago) and hence, are more perfect than those made by the ordinary method. While the points of these instruments of Mr. S. have received very especial attention in every particular, the handles, we think, are the neatest we have seen. They are six-sided, slightly tapering, finely polished, and with a deep spring temper blue.

There is a charm about beautiful things. We are delighted with the progress—we had almost said the high degree of perfection, attained by some of the manufacturers of our dental instruments, especially those we have named. We are always glad to announce such things to our professional brethren, and should account that we are recreant to their best interests if we refused to do. T.

